# Retrospective Analysis of Factors Associated with Fracture in 714 Patients with Polymyalgia Rheumatica

**DOI:** 10.1155/2022/9409883

**Published:** 2022-02-12

**Authors:** Rajiv Ark, Khojasta Talash, Marwan Bukhari

**Affiliations:** ^1^Royal Lancaster Infirmary, University Hospitals of Morecambe Bay NHS Foundation Trust, Lancaster, UK; ^2^Lancaster University Medical School, Lancaster University, Lancaster, UK

## Abstract

**Introduction:**

Polymyalgia rheumatica (PMR) is a disease of the elderly, associated with increased fracture risk due to glucocorticosteroid (GC) treatment with the additional possible influence of chronic inflammation. Risk factors for fracture in PMR have not been extensively studied. Hip structure analysis (HSA) is a way to measure bone morphology in the hip using dual X-ray absorptiometry (DEXA). It has been used as a predictor of fracture in epidemiological settings. HSA has not been studied in PMR before.

**Objectives:**

The object of this retrospective study was to determine if fracture risk in PMR was associated with densitometry data and to determine the influence, if any, of HSA on that association.

**Methods:**

714 patients with PMR referred for a bone density estimate at a district general hospital from June 2004 to October 2010 were studied. Demographic data, GC use, alcohol consumption, smoking status, secondary osteoporosis, and fracture history were recorded. Bone mineral density (BMD), *Z* score, *T* score, body composition data, and HSA measurements were collected. These were geometric measurements taken from 2-dimensional DEXA images of the hip. Fracture was modelled as an outcome variable using logistic regression models, adjusted for age and sex. And the fit of the model was assessed by comparing the area under the curve (AUC).

**Results:**

714 patients were studied, 532 (75%) were female, and mean age was 70.5 with SD of 8.8. 703 (98%) had been treated with GCs. Lumbar and femoral BMD models were significantly associated with fracture. Right femur OR 0.062 (0.014-0.285), left femur OR 0.098 (0.023-0.412), right femoral neck 0.078 (0.014-0.43), left femoral neck 0.104 (0.022-0.492), L1 0.192 (0.066-0.56), L2 OR 0.138 (0.053-0.358), L3 0.192 (0.079-0.463), and L4 0.243 (0.108-0.544). Cross-sectional area was the only HSA parameter that was associated with fracture OR 0.988 (0.980–0.997).

**Conclusion:**

L2 association models were strongest. Prospective studies are needed to elucidate whether these factors predict future fracture. GC data were binary, not reflecting dose and duration.

## 1. Introduction

Polymyalgia rheumatica (PMR) is a chronic inflammatory condition that commonly affects the elderly resulting in pain in the shoulders and hips. It is associated with an increased risk of fracture in the UK population, with a hazard ratio of 1.63 and 95% confidence interval of 1.54–1.73 [1]. The most common site of fractures is the vertebrae, and they can happen despite antiresorptive therapy [2]. Fractures can lead to chronic pain and reduced quality of life.

The British Society of Rheumatology (BSR) guidelines recommend treatment with prednisolone and coprescription of calcium and vitamin D supplementation, acknowledging the risk of glucocorticoid-induced osteoporosis [3]. Duration of glucocorticosteroid (GC) treatment can be months and even years until fully discontinued [4]. Oral glucocorticoid use can lead to a reduction in BMD [5]. Cumulative GC dose in PMR has been linked to increased fracture risk in some studies [6]; however, others dispute this [4].

The pathogenesis of PMR is not fully understood; however, inflammation has been demonstrated in the synovium of shoulder and hip joints [7]. Chronic inflammation may increase fracture risk independent of glucocorticoid use [8]. Inactivity due to pain is linked to low bone mineral density and has been implicated in PMR fractures [9]. PMR is also associated with giant cell arteritis (GCA); in patients diagnosed with GCA and PMR, the risk of fracture is increased further [1].

The risk factors for fracture in PMR have not been extensively studied. A study of a South Korean cohort found that trabecular bone score predicted fracture; however, lumbar spine BMD did not [10]. Erythrocyte sedimentation rate (ESR) and visual analogue scale (VAS) score have also been used to predict fracture [8]. DEXA scans provide data about bone mineral density from which *T* scores and *Z* scores can be derived. *T* score values at the femoral neck have been validated in predicting the risk of fracture with other factors from the patient's medical and social history as part of the FRAX tool [11].

Hip structural analysis (HSA) is a technique that uses measurements from DEXA images to assess the hip bone structure. Geometric parameters taken from these images have been used to predict fractures in osteoporosis [12]; however, its use has not been validated in patients with PMR.

HSA has been shown to predict fracture independently of bone mineral density in postmenopausal women [13, 14]. HSA parameters are unfavourable in children with certain metabolic conditions such as girls with type 1 diabetes mellitus and boys with anorexia nervosa leading to increased fracture risk [15, 16].

This study was aimed at identifying factors that were associated with fractures in patients with PMR, including HSA, which has not been studied before to our knowledge.

## 2. Methods

This was a retrospective cohort study of patients with PMR who underwent routine DEXA scans.

Data were collected from patients referred for DEXA scans at the University Hospitals of Morecambe Bay NHS Foundation Trust in Lancaster, UK. Data were collected from scans between June 2004 and October 2010. All patients must have had a clinical diagnosis of PMR by the referrer at the time of the scan to be included. The study was approved by the North West Regional Ethics Committee.

The outcome variable studied was whether the patient had sustained a fragility fracture at any site before the scan date. Other variables studied included demographic data such as age at the time of scan and sex of the patient. Alcohol consumption and smoking history were also recorded.

Height and weight were measured and used to calculate body mass index (BMI). It was recorded if the patient had previous treatment with GCs and further if the patient was still on current treatment with GCs. Treatment with calcium and vitamin D supplementation for GC induced osteoporosis prophylaxis was recorded. It was recorded if the patient had a concurrent diagnosis of rheumatoid arthritis before the scan date. The presence of a diagnosis of secondary osteoporosis before the scan date was also recorded. This was defined as the presence of a disorder strongly associated with osteoporosis as outlined in the FRAX tool [11].

These data were collected by the professional performing the scan and stored in a database using Microsoft Access (Microsoft Corporation, 2017). DEXA machine and parameters were calibrated before imaging. Fat mass, bone mass, and average tissue thickness were calculated from DEXA images.

Bone mineral density was calculated from DEXA images of the left and the right femur and femoral necks. They were also calculated for lumbar spine vertebrae L1, L2, L3, and L4. *T* score was calculated from the bone mineral density at each level. *Z* score was calculated for each level using the bone mineral density and age at the date of scan and sex.

**Figure 1:**
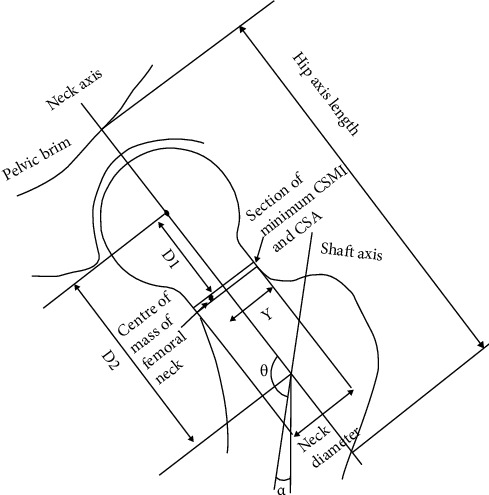
Diagram showing different geometric parameters measured from 2-dimensional DEXA images in hip structural analysis.

Hip structural analysis measurements were taken from DEXA scan images. The hip axis length (HAL) was the distance along the femoral neck axis from the base of the trochanter to the pelvic brim. The cross-sectional area (CSA) of the femoral neck was calculated. Cross-sectional moment of inertia (CSMI), which represented the bending rigidity of the femoral neck, was also calculated. The femoral shaft angle (alpha) and the neck/shaft angle (theta) were measured. *Y* was the distance from the centre of mass of the femoral neck to the superior neck margin. Strength index (SI) was an indicator of proximal femur strength. D1 was the distance from the centre of the femoral head to the centre of the femoral neck. D2 was the distance from the centre of the femoral head to the intertrochanteric line. D3 was the mean femoral neck diameter. These measurements are illustrated in Figure 1.

Statistics were carried out using “R: a Language and Environment for Statistical Computing” (2019. Vienna, Austria). Demographic characteristics were compared using Fisher's exact test for categorical variables and Student's *T*-tests for continuous variables. The fracture was modelled as an outcome variable in multivariate binomial logistic regression models, adjusting for age and sex. Odds ratios (OR) with 95% confidence intervals were calculated. The area under the receiver operating characteristic curve (AUC) for significant association models was calculated to estimate the goodness of fit.

In a previous population-based study, the prevalence of fragility fracture was estimated to be present in 13.9% of PMR patients [1]. The sample size needed was estimated to be 666 given a power of 0.95 and alpha error probability of 0.05 to detect an increase in fracture risk of 5%.

## 3. Results

714 patients with PMR who underwent a DEXA scan between June 2004 and October 2010 were studied. Of the 714 patients in this study, 156 (21.8%) had sustained a fracture.

### 3.1. Descriptive Data

Descriptive data of patients with PMR with and without fracture are shown in Table 1. 532 patients were female (74.5%). Of the patients with a fracture, 139 (89.1%) were female, whereas 393 (70.4%) of the patients without a fracture were female. 713 patients were White Caucasian, and 1 was of Asian ethnicity. The mean age of patients in this cohort was 70.5 years, 72.5 in patients with fractures and 70.0 in patients without fractures. 35 (6.3%) of nonfracture patients consumed alcohol, whereas 4 (2.6%) of patients with a fracture did. 198 (35.5%) of nonfracture patients smoked, whereas 57 (36.5%) of fracture patients smoked.

549 (98.4%) of nonfracture patients had been treated with GCs and 154 (98.7%) of fracture patients. 476 patients (85.3%) were on current GC treatment in nonfracture patients compared to 123 (78.8%) in fracture patients; this was significantly fewer (*p* = 0.03). 275 (49.3%) of nonfracture patients were on GC-induced osteoporosis prophylaxis compared to 90 (57.7%) in fracture patients (*p* = 0.07). 45 (8.1%) nonfracture patients had developed secondary osteoporosis, whereas 22 (14.1%) fracture patients had; this was significantly higher in patients with a history of fracture (*p* = 0.04).

### 3.2. Binomial Logistic Regression Models

Odds ratios of fracture and 95% confidence intervals for BMD, *T* score and *Z* score at each level, and AUCs of models are shown in Table 2. Fractures were more common in females, with OR of 3.43 and 95% confidence interval (2.01-5.86). Fractures were also more likely with increasing age, with OR of 1.03 (1.01-1.05).

Bone mineral density, *T* score, and *Z* score were associated with fracture using measurements from the left and the right femur, femoral necks, and L1, L2, L3, and L4. AUC was highest in L2 models.

History of smoking was not associated with fracture, with OR of 1.21 (0.82-1.77), and neither was alcohol consumption with 0.64 (0.22-1.90) or family history of fracture with 1.45 (0.71-2.95).

Previous GC use was not associated with fracture of 1.20 (0.25-5.89) or current GC use of 0.70 (0.43-1.14). Secondary osteoporosis was associated with fracture in univariate analysis of 1.87 (1.09-3.23); however, when adjusted for age and sex, it was nonsignificant at 1.38 (0.79-2.41).

BMI was not associated with fracture: OR of 1.002 (0.968-1.037), and neither was the average tissue thickness OR of 1.019 (0.954-1.088). Fat mass was not a significant associated with fracture OR of 1.000 (0.998 - 1.002) and neither was the lean mass: 1.000 (0.998-1.002).

### 3.3. Hip Structural Analysis

Odds ratios of fracture for different HSA parameters are shown in Table 3. CSA was associated with fracture risk; OR was 0.988 with a 95% confidence interval of 0.980-0.997. The AUC for the CSA regression model was 0.6739. HAL was not associated with fracture, with an OR of 1.008 (0.982-1.035), and neither was CSMI, with an OR of 1.000 (0.999-1.000). D1 was not associated with fracture, with an OR of 1.029 (0.972-1.089), and neither was D2, with an OR of 1.010 (0.981-1.040), nor D3, with an OR of 1.033 (0.962-1.109). Alpha was not associated with fracture, with an OR of 0.983 (0.940-1.029), and neither was theta, with an OR of 1.007 (0.975-1.039). SI was not associated with fracture in regression models, with an OR of 0.683 (0.406-1.150), and neither was *Y*, with an OR of 1.087 (0.966-1.223).

## 4. Discussion

The main significant finding is that BMD at the lumbar spine was associated with fracture in PMR patients. To our knowledge, this has not been demonstrated before. In a smaller South Korean cohort, lumbar bone mineral density was observed not to predict fracture in PMR patients [10]. L2 models had the highest AUCs for association with fracture. Although hip pain is a more characteristic symptom of PMR, and inflammation has been observed in the synovium of proximal joints, lumbar BMD was also affected. However, vertebral fractures are the most common site of fracture in PMR [2]; therefore, BMD models in this area should still be considered.

HSA measurements in PMR have not previously been associated with fracture. HSA parameters have been linked to increased fracture risk in postmenopausal women, boys with anorexia nervosa and girls with T1DM. CSA was significantly associated with fracture risk; this geometric measurement has been linked to the strength of the bone and the likelihood of fracture [12]. Although this is statistically significant, the effect size is small with an OR of 0.988 and may not be clinically helpful. Nevertheless, as it is a geometric measurement independent of BMD, it could help improve the accuracy of predictor models. Other HSA measurements were not associated with fracture. These are indicators of the structural strength of the hip specifically, and most fractures in PMR patients occur in the vertebrae.

BMD was associated with fracture in the femurs and to a lesser extent the femoral necks too.

*T* score models had slightly higher AUCs, and *Z* score models had even higher AUCs. Perhaps a reason for the reduced effect size in ORs of *T* and *Z* scores over BMD is that these are calculated by comparing against large databases. BMD values used for the regression models were only compared against the PMR patients in this cohort [17].

Previous treatment with GCs was not linked to increased fracture risk. A higher proportion of PMR patients without fracture were noted to be on current GC treatment; however, when adjusted for age and sex, there was no association between current GC treatment and fracture. Nevertheless, this might imply that stopping GC treatment is more prevalent in fracture patients or, conversely, that nonfracture patients may be overtreated and could be tapered more quickly. In the general population, GC use can lead to secondary osteoporosis and, in turn, increase the risk of fracture [5]. Timing of fracture and GC therapy was not recorded; therefore, it is not possible to tell how many patients had a fracture or had osteoporosis before starting GC therapy. There was no significant difference in the proportion of patients on GC-induced prophylaxis in the two groups; however, the timing of initiation of prophylaxis was not known. It was not recorded if patients were treated with bisphosphonates to protect against this, which may have reduced the effect of this in our cohort. While some studies imply GCs have a role in the pathogenesis of corticosteroid-induced osteoporosis, others do not [4, 6]. One study even noted that fractures were prevalent before GC treatment was started [9].

GC data were also collected as binary data. Although it differentiated whether a patient was on current treatment or had been on previous treatment, it did not reflect dose or duration. GC use in PMR can vary depending on how long symptoms persist. Cumulative GC dose has been used to predict fracture risk.

In univariate analysis, diagnosis of secondary osteoporosis was associated with increased fracture risk; however, when adjusted for age and sex, it was not significant. These data did not allow adjustment for maximum dose or duration of GC treatment which is a major weakness of the study.

These data suggest that fractures were more likely to occur in females. This can also be observed in the general population as females over 50 are more than twice as likely to fracture than males [18]. This is likely to be due to menopause-related oestrogen deficiency [19]. The OR of fracture in females compared to males was higher than expected at 3.43. This may be due to increased inflammatory changes in females with PMR compared to males. Females with PMR require more GCs than males and have a less marked decrease in ESR compared to males [20]. There was an increased risk of fracture with increasing age; this is also observed in the general population [21].

Alcohol consumption was not associated with fracture in this cohort; however, in the general population, it is linked to an increased risk of fracture [22]. A potential reason for this is that alcohol consumption was collected as binary data. At small amounts of alcohol consumption, there is no effect on fracture risk [22].

Smoking was also not associated with increased fracture risk. Current smoking has been associated with increased fracture risk compared to a history of smoking; however, our study did not differentiate between the two [23]. The risk ratio is larger in males [23]; with a mainly female cohort, this study may have been underpowered to detect a difference.

BMI was not associated with fracture in our cohort of PMR patients. BMI has been observed to have a nonlinear risk of fracture, particularly for hip fracture [24]. Fat mass and lean mass was not associated with fracture. Skeletal lean mass has been associated with increased BMD [25]; however, BMD itself proved to have a better association.

Average tissue thickness was not associated with fracture. Increased soft tissue thickness has been hypothesized to reduce fracture risk by attenuating forces to the femur during a fall [26]. This does not appear to be the main mechanism or site of fracture in the PMR cohort.

This study was limited in design as it was retrospective and only showed association to previous fractures. This study represents a selection of PMR patients because only patients with a suspicion of osteoporosis are referred for a DEXA scan. A population-based study estimated the prevalence of PMR to be 0.85% [27]; extrapolating this figure for the catchment area population of 370,000, we would estimate the number of PMR patients to be around 3,145.

Other potential confounders not adjusted for were level of activity and GCA diagnosis.

ESR and VAS are scales that could also predict fracture alongside information from DEXA scans.

We relied on GP diagnosis of PMR and to supply accurate additional information which feeds into the FRAX tool. ACR/EULAR classification criteria for PMR were published in 2012, which may have reclassified patients from whom information was collected before this date. Data were collected from one district general hospital in the UK, with a population of mainly one ethnic group; this is characteristic of the local population in the age group. These results may not be generalizable across other parts of the country and ethnicities.

In conclusion, lumbar BMD is significantly associated with fracture in patients with PMR, which has not previously been demonstrated. DEXA measurements of the spine should be considered as well as hip measurements when assessing PMR patients for fracture risk. HSA, which has not been studied in association with fracture in PMR patients, was not associated with fracture, apart from CSA, although OR indicated a modest effect size. Further prospective work is needed to characterize how useful lumbar BMD is at predicting a fracture in the future.

## Figures and Tables

**Table 1:** Descriptive data of patients with polymyalgia rheumatica with and without fracture.

	No fracture	Fracture	*p* value
Total	558	156	
Male, *n* (%)	165 (29.6%)	17 (10.9%)	<0.01^∗^
Age, mean (std. dev)	72.5 (8.9)	70.0 (8.7)	<0.01^∗^
RA	19 (3.4%)	3 (1.9%)	0.44
BMI	28.2 (5.2)	28.3 (5.3)	0.95
Alcohol	35 (6.3%)	4 (2.6%)	0.08
Smoker	198 (35.5%)	57 (36.5%)	0.85
Previous steroid use	549 (98.4%)	154 (98.7%)	1
Secondary osteoporosis	45 (8.1%)	22 (14.1%)	0.03^∗^
Current steroid use	476 (85.3%)	123 (78.8%)	0.04^∗^

∗ indicates that the parameter is a significantly associated, *p* < 0.05. Secondary osteoporosis is defined as the presence of a disorder strongly associated with osteoporosis as outlined in the FRAX tool.

**Table 2:** Odds ratios of fracture and 95% confidence intervals for BMD, *T* score, and *Z* score at each level and AUCs of models. Binomial logistic regression models were adjusted for age and sex.

	BMD	AUC	*T* score	AUC	*Z* score	AUC
Left femur	0.098 (0.023, 0.412)	0.682	0.728 (0.607, 0.873)	0.694	0.677 (0.552, 0.831)	0.694
Right femur	0.062 (0.014, 0.285)	0.692	0.713 (0.593, 0.858)	0.692	0.662 (0.538, 0.815)	0.693
Left femoral neck	0.104 (0.022, 0.492)	0.673	0.738 (0.6, 0.908)	0.683	0.703 (0.56, 0.881)	0.685
Right femoral neck	0.078 (0.014, 0.43)	0.684	0.734 (0.597, 0.902)	0.684	0.694 (0.553, 0.871)	0.684
L1	0.192 (0.066, 0.56)	0.679	0.82 (0.716, 0.94)	0.692	0.798 (0.688, 0.924)	0.691
L2	0.138 (0.053, 0.358)	0.698	0.787 (0.697, 0.888)	0.710	0.763 (0.669, 0.871)	0.711
L3	0.192 (0.079, 0.463)	0.688	0.823 (0.735, 0.921)	0.696	0.805 (0.713, 0.908)	0.696
L4	0.243 (0.108, 0.544)	0.684	0.852 (0.768, 0.945)	0.691	0.837 (0.749, 0.934)	0.691

**Table 3:** Odds ratios of fracture for different HSA parameters.

HSA parameter	Odds ratio (95% confidence interval)
HAL	1.008 (0.982–1.035)
CSMI	1.000 (0.999–1.000)
CSA	0.988 (0.980–0.997)^∗^
D1	1.029 (0.972–1.089)
D2	1.010 (0.981–1.040)
D3	1.033 (0.962–1.109)
*Y*	1.087 (0.966–1.223)
Alpha	0.983 (0.940–1.029)
*Θ*	1.007 (0.975–1.039)
SI	0.683 (0.406–1.150)

∗ indicates parameter is significantly associated, *p* < 0.05. HAL: hip axis length; CSMI: cross-sectional moment of inertia; CSA: cross-sectional area; D1: distance from the centre of the femoral head to the centre of the femoral neck; D2: distance from the centre of the femoral head to the intertrochanteric line; D3: mean femoral neck diameter; *Y*: distance from the centre of mass of the femoral neck to the superior neck margin; Alpha: femoral shaft angle; *Θ*: neck shaft angle; SI: strength index (composite measure).

## Data Availability

The data used to support the findings of this study are restricted by the North West Regional Ethics Committee in order to protect patient privacy. Data are available from Dr. Marwan Bukhari, Royal Lancaster Infirmary, for researchers who meet the criteria for access to confidential data.
